# The arginine-ornithine antiporter ArcD contributes to biological fitness of *Streptococcus suis*

**DOI:** 10.3389/fcimb.2014.00107

**Published:** 2014-08-12

**Authors:** Marcus Fulde, Joerg Willenborg, Claudia Huber, Angela Hitzmann, Daniela Willms, Maren Seitz, Wolfgang Eisenreich, Peter Valentin-Weigand, Ralph Goethe

**Affiliations:** ^1^Department of Infectious Diseases, Institute for Microbiology, University of Veterinary MedicineHannover, Germany; ^2^Department of Medical Microbiology, Helmholtz Centre for Infection Research (HZI)Braunschweig, Germany; ^3^Lehrstuhl für Biochemie, Technische Universität MünchenGarching, Germany

**Keywords:** *Streptococcus suis*, zoonosis, arginine-ornithine antiporter, biological fitness, arginine deiminase system

## Abstract

The arginine-ornithine antiporter (ArcD) is part of the Arginine Deiminase System (ADS), a catabolic, energy-providing pathway found in a variety of different bacterial species, including the porcine zoonotic pathogen *Streptococcus suis*. The ADS has recently been shown to play a role in the pathogenicity of *S. suis*, in particular in its survival in host cells. The contribution of arginine and arginine transport mediated by ArcD, however, has yet to be clarified. In the present study, we showed by experiments using [U-^13^C_6_]arginine as a tracer molecule that *S. suis* is auxotrophic for arginine and that bacterial growth depends on the uptake of extracellular arginine. To further study the role of ArcD in arginine metabolism, we generated an *arc*D-specific mutant strain and characterized its growth compared to the wild-type (WT) strain, a virulent serotype 2 strain. The mutant strain showed a markedly reduced growth in chemically defined media supplemented with arginine when compared to the WT strain, suggesting that ArcD promotes arginine uptake. To further evaluate the *in vivo* relevance of ArcD, we studied the intracellular bacterial survival of the *arc*D mutant strain in an epithelial cell culture infection model. The mutant strain was substantially attenuated, and its reduced intracellular survival rate correlated with a lower ability to neutralize the acidified environment. Based on these results, we propose that ArcD, by its function as an arginine-ornithine antiporter, is important for supplying arginine as substrate of the ADS and, thereby, contributes to biological fitness and virulence of *S. suis* in the host.

## Introduction

*Streptococcus* (*S*.) *suis* is a frequent colonizer of mucosal surfaces of the upper respiratory and the gastrointestinal tract in pigs. As a facultative pathogen, *S. suis* is able to cross epithelial barriers and induce a variety of fatal diseases, such as meningitis, septicaemia, arthritis, and bronchopneumonia. Thus, high economic losses characterize *S. suis* as one of the most important agents in pig breeding and pork processing industries (Clifton-Hadley and Alexander, 1980; Arends and Zanen, 1988; Chanter et al., 1993; Staats et al., 1997; Swildens et al., 2004; Fulde and Valentin-Weigand, 2013).

*S. suis* is receiving increasing attention as a zoonotic agent due to outbreaks in China in 1998 and 2005. Noteably, *S. suis* is currently considered as the most frequent cause of adult bacterial meningitis in Vietnam (Tang et al., 2006; Yu et al., 2006; Mai et al., 2008; Wertheim et al., 2009). Furthermore, recent reports from different countries of human infections indicate a growing awareness of *S. suis'* zoonotic potential (Lun et al., 2007). However, despite of its increasing importance, pathogenesis of *S. suis* infections in humans and pigs including knowledge on bacterial virulence factors and host responses is far from being understood (Fulde and Valentin-Weigand, 2013).

One of first virulence-associated traits discovered for *S. suis* was the arginine deiminase system (ADS). The ADS comprises an enzymatic pathway converting arginine to citrulline with the concomitant production of ornithine, ammonia, carbon dioxide, and ATP (Cunin et al., 1986; Barcelona-Andrés et al., 2002). The wide distribution of the ADS among all kingdoms of life and the high conservation of genes and their arrangement supports its outstanding role as a secondary, energy providing pathway (Gruening et al., 2006). The ADS has a pivotal role in the pathogenicity of many bacteria, such as *Streptococcus* spp., *Listeria monocytogenes, Staphylococcus* spp. and parasites, such as *Giardia lamblia*. Under acidic conditions, e.g., in the phagolysosome of host cells, the ammonium produced by the ADS-dependent arginine catabolism is sufficient to significantly prolong the intracellular survival of the bacteria (Casiano-Colon and Marquis, 1988; Curran et al., 1995; Degnan et al., 1998; Benga et al., 2004; Ryan et al., 2009; Fulde et al., 2011; Cheng et al., 2013; Lindgren et al., 2014). In addition, the arginine deiminase ArcA and the antimicrobial host protein inducible NO-synthase (iNOS) compete for the same substrate as shown for the intestinal pathogen *G. lamblia* (Ringqvist et al., 2008).

The important role of the ADS in metabolism and pathogenesis suggests a tight regulation by a complex regulatory network which responds to a variety of different environmental stimuli. Indeed, type and amount of carbon sources, oxygen tension, substrate (arginine) availability and temperature have been shown to directly or indirectly influence ADS expression (Winterhoff et al., 2002; Dong et al., 2004; Gruening et al., 2006; Zeng et al., 2006; Makhlin et al., 2007; Liu et al., 2008; Ryan et al., 2009; Fulde et al., 2011; Willenborg et al., 2011, 2014; Hitzmann et al., 2013). In *S. suis*, the transcriptional regulator ArgR is highly specific for the regulation of ADS, underlining a particular relevance of arginine and its catabolism by the ADS for the metabolism of *S. suis* (Fulde et al., 2011). Two genes with significant homologies to an arginine-ornithine antiporter (*arc*D) and a putative Xaa-His dipeptidase (*arc*T) are associated with the ADS of some bacteria including streptococci. In *Pseudomonas aeruginosa*, ArcD is a transmembranal protein composed of 13 helices (Lüthi et al., 1990; Verhoogt et al., 1992; Bourdineaud et al., 1993). Similar to ArcD of *Lactococcus* (*Streptococcus*) *lactis*, ArcD facilitates an ATP-independent, electro-neutral exchange of arginine and ornithine across the bacterial membrane, thereby providing a substrate for ADS mediated arginine catabolism (Driessen et al., 1987; Verhoogt et al., 1992; Bourdineaud et al., 1993). The putative function of ArcD as an arginine-ornithine antiporter was also shown by Wimmer et al. for the archaeon *Halobacterium salinarum* (Wimmer et al., 2008). On the other hand, an involvement of ArcD in virulence is yet speculative. A recent publication by Gupta et al. (2013) showed that an *arc*D-deficient *S. pneumoniae* mutant was attenuated in murine models of pneumonia and bacteraemia. However, whether or not the *arc*D gene of *S. pneumoniae* functions as an arginine-ornithine antiporter remained unanswered.

The present study focused on ArcD of *S. suis*. Determination of extracellular arginine and intracellular ornithine confirmed an involvement of ArcD in arginine uptake. Subsequent phenotypic characterization of an isogenic *arc*D-deficient mutant strain revealed a significant attenuation in terms of biological fitness and survival under acidic conditions.

## Materials and methods

### Bacterial strains and growth conditions

*S. suis* strain 10 (Smith et al., 1999), a highly virulent serotype 2 strain, was used in this study. Bacteria were routinely grown on blood agar plates (BD) at 37°C with 5% CO_2_, or cultivated in liquid TSB (BD, Heidelberg, Germany) medium under the same conditions. Following day, bacteria were adjusted to an optical density at 600 nm of 0.05 in a tryptone-yeast-based medium supplemented with 10 mM glucose or galactose, respectively. If indicated, 50 mM arginine was supplemented (Burne et al., 1987; Zeng et al., 2006). Auxotrophy studies were performed in a chemically defined medium (CDM) in the presence of absence of arginine essentially as described elsewhere (van de Rijn and Kessler, 1980; Hitzmann et al., 2013). Growth was monitored every hour using a Nova Spec II Photometer (Pharmacia, Freiburg, Germany). Assays were performed in triplicates and repeated at least four times.

To determine the transcriptional organization of the ADS, bacteria were grown in TY medium supplemented with 50 mM arginine and 10 mM glucose or galactose, respectively, to an OD_600_ of 0.2. Then, 10 ml of bacterial culture were harvested by centrifugation. Pellets were resuspended in 1 ml of Trizol (Invitrogen/Life Technologies, Carlsbad, California, USA) and immediately snap-frozen in liquid nitrogen.

### DNA and RNA techniques, cDNA synthesis and reverse transcriptase PCR

If not stated otherwise, all enzymes and reagents were purchased from Invitrogen (Life Technologies, Carlsbad, California, USA) and NEB (New England Biolabs, Frankfurt am Main, Germany). Chromosomal DNA was prepared using the Qiagen's DNeasy Blood and Tissue Kit (Qiagen, Hilden, Germany) according to the manufacturer's recommendations. Plasmid DNA was purified with the NucleoSpin® Plasmid Kit (Macherey-Nagel, Dueren, Germany) according to manufacturer's instructions. RNA was prepared as described by Hitzmann et al. (2013) using the Ambion's RiboPure™-Bacteria Kit. Residual DNA was digested with the Ambion TURBO DNA-free™-kit. Complementary DNA (cDNA) was synthesized from 1 μg total RNA using random primers (3 μg). Primers and RNA were heated for 10 min at 70°C in 12 μl of dH2O and then chilled on ice. Eight μl of master mix, consisting of 4 μl 5x first strand buffer, 2 μl 10 mM dNTP mix, 1 μl RNase Inhibitor and 1 μl 100 mM DTT, were added and incubated for 5 min at 25°C. Then, 1 μl of Reverse Transcriptase (SuperScript II) was added and another 10 min incubation-step at 25°C followed. Then the reaction was incubated at 42°C for 1 h and the Reverse Transcriptase was inactivated. cDNA was purified using the Qiagen PCR-purification kit. To analyse the transcriptional organization of the ADS, RT-PCR was performed using following intergenic primer pairs: *flp*S-*arc*A (CGA TGG TCT TGT TTG AAA CCT/ACA CCA GCC ATC GTT TTC TC), *arc*D-*arc*T (CTC CAC ATG GGT GAA GAA GG/CGC CAT CGA AGG ACC TTT A), *arc*T-*arc*H (CTG CGG ATA AAG AAG CCC TA/CTG ATG CTG GCT GTT GGT TA).

### Mutagenesis

ArcD was inactivated by insertion mutagenesis as described earlier (Fulde et al., 2011). Briefly, the gene *arc*D was amplified from the streptococcal genome with the primers *arc*DKOfor (CCG TTA CTG TGG CTG AAT TGG) and *arc*DKOrev (CCT TGC AAT CCT TCT TCA CC) and subsequently introduced into the cloning vector *p*GEM®-T Easy (Promega, Mannheim, Germany). The resulting plasmid *p*GEM-*arc*D was linearized by *Hpa*I. Then, the *Pvu*II released erythromycin resistance cassette derived from vector *p*IC*erm* (kindly provided by Christoph Baums, Institute for Microbiology, University of Veterinary Medicine, Hannover) was introduced to disrupt *arc*D. Electroporation was essentially performed as previously described (Smith et al., 1995). Mutants were tested for integrity by PCR using the primer pair *arc*DKOfor/*arc*DKOrev.

### Quantification of arginine

A method to determine arginine in the supernatant of bacterial cultures was developed based on the Sakaguchi reaction (Sakaguchi, 1925). Briefly, bacteria were grown in TY medium in the presence of 10 mM arginine. At an OD_600_ of 0.2, streptococci were harvested by centrifugation and the resulting supernatant was filtrated using the Millex® Syringe Filters with pore size of 0.22 μm (Merck Millipore, Schwalbach, Germany). Then, 100 μl of bacterial supernatant was mixed with 100 μl reagent A (0.05% (w/v) chloronaphthol, 5% urea (m/v) in 95% EtOH). After extensive shaking, 200 μl reagent B (0.7% Brom (v/v), 5% NaOH (w/v) in H_2_O) was added. A change in color was determined spectro-photometrically by an OD of 500 nm. Quantification was done along a calibration curve with different concentrations of arginine diluted in TY medium. Non-inoculated TY medium served as control.

### Labeling experiments using [U-^13^C_6_]arginine

In all labeling experiments, a CDM overnight culture of the indicated *S. suis* strain was harvested by centrifugation, washed twice in PBS, and then inoculated in fresh CDM to an OD_600_ of 0.002. In experiments determining the ^13^C-label in proteinogenic amino acids, bacteria were grown in CDM containing 2.5 mM [U-^13^C_6_]arginine (Campro Scientific) at 37°C and harvested at an OD_600_ of 0.2 by centrifugation at 4000 × g at 4°C for 5 min. The bacterial cells were washed twice in ice-cold PBS, immediately autoclaved at 120°C for 15 min, and lyophilized. Samples were hydrolyzed under acidic conditions. The resulting amino acids were purified using a cation exchange column, converted into TBDMS derivatives (except for arginine, see below), and analyzed by GC/MS as described earlier (Eylert et al., 2008). For the [U-^13^C_6_]arginine uptake experiments, bacteria were first grown in CDM with ^12^C-arginine to an OD_600_ of 0.2, then harvested by centrifugation, washed twice in PBS, and afterwards transferred to CDM without any arginine to induce arginine starvation for 15 min at 37°C. The bacteria were concentrated by centrifugation and then incubated in CDM containing 2.5 mM [U-^13^C_6_]arginine at 37°C for 30 min. Bacteria were then pelleted by centrifugation, washed twice in ice-cold double-distilled water, and immediately disrupted by ultrasonic disintegration in a Branson Sonifier with continuous water cooling for 15 min at 4°C, and output control of 8. The lysates were cleared by centrifugation at 10,000 × g at 4°C for 30 min and the remaining supernatant lyophilized for further analysis.

### Arginine and ornithine determination

Arginine does not form trimethylsilyl (TMS) and tertbutyldimethylsilyl (TBDMS) derivatives (Halket et al., 2005) and can be analyzed as arginine- trifluoroacetic acid (TFA)-methylester (Darbre and Islam, 1968). An aliquot of the cation exchange eluate described above was dried under a stream of nitrogen and dissolved in 200 μl of methanolic HCl (3N). The mixture was heated to 70°C for 30 min and then dried under a stream of nitrogen. The residue was dissolved in 50 μl of TFA and heated to 140°C for 10 min. The mixture was dried again, dissolved in 100 μl of anhydrous ethylacetate and subjected to GC/MS analysis. General GC/MS conditions were the same as described for amino acid TBDMS derivatives (Eylert et al., 2008). For TFA-methylester derivatives the column was kept at 70°C for 3 min and then developed with a temperature gradient of 10°C min^−1^ to a final temperature of 200°C that was kept for 3 min. The retention time for the arginine-TFA-methylester was 17.2 min. The molecular mass of the arginine derivative was 476. ^13^C-excess calculations were performed with m/z 407 [M-CF_3_]^+.^, a fragment still containing all C atoms of arginine.

Ornithine was determined from the freeze dried supernatant and derivatized as described for arginine. Under identical GC/MS conditions, ornithine was analyzed as ornithine-TFA-methylester (M: 338) at R_*t*_ 14.0 min. The observed fragment m/z 306 corresponds to [M-CH_3_OH]. The ^13^C/^12^C ratio was calculated with the relative intensities of m/z 306 (^12^C-ornithine) and m/z 311 ([U-^13^C_5_]ornithine).

### Determination of pH and ammonia in the culture supernatant

Determination of ammonia in the culture supernatant of WT strain 10 and its *arc*D-deficient mutant strain was performed using the ammonia assay kit (Sigma, Munich, Germany) as described previously (Fulde et al., 2011). pH values in the bacterial culture supernatant were determined using a specific electrode (pH 197, WTW, Weilheim, Germany).

### Determination of arginine deiminase (AD) activity

AD activity was determined according to the protocol of Oginsky (1957) and Degnan et al. (1998) as described previously (Gruening et al., 2006; Winterhoff et al., 2002). Briefly, bacteria were grown in CDM medium as in the [U−^13^C_6_]arginine uptake assays and harvested by centrifugation. Then, bacteria were lysed and the respective lysates were incubated for 2 h in a 0.1 M potassium phosphate buffer containing 10 mM L-arginine at 37°C. The supplementation of 250 μl of an acidic solution (1:3, 96% sulfuric acid and 85% orthophosphoric acid) stopped enzymatic reactions. After addition of 31.3 μl of a 3% diacetyl monoxime solution, the suspension was incubated for 15 min at 100°C. Production of citrulline was determined colorimetrically at an OD_450_. Results are given in nmol citrulline produced in 1 h per mg whole cell protein

### Bacterial survival under acidic conditions

Experiments were performed essentially as described earlier (Benga et al., 2004; Gruening et al., 2006). Briefly, WT strain 10 and its isogenic mutant strain 10Δ*arc*D were grown overnight in TSB. Then, bacteria were harvested by centrifugation and resuspended in a buffer containing 20 mM Na_2_HPO_4_, 1 mM MgCl_2_, 25 mM arginine-HCl adjusted to pH 5, 6, or 7, respectively. Bacteria were incubated at 37°C for the indicated intervals and survival was monitored by plating. Results represent means and standard deviations of one experiment performed in triplicates. Experiments were repeated at least three times.

### Intracellular survival of *s. suis* in HEp-2 cells

The ability of the wild-type strain 10 and the *arc*D deficient mutant strain to survive in HEp-2 epithelial cells was determined as described previously with some modifications (Benga et al., 2004; Fulde et al., 2011). Briefly, in addition to untreated HEp-2 cells, parallel assays were done with HEp-2 cells that had been pre-treated with bafilomycin (200 nM) for 1 h to inhibit endosomal acidification. HEp-2 cells were then infected with 100 bacteria per cell (MOI 100:1) for 2 h and afterwards washed thrice with PBS. In parallel, cells were incubated in DMEM containing 31.25 μg ml^−1^ Daptomycin (Cubicin®) for 90 and 210 min, respectively, at 37°C with 8% CO_2_ to kill extracellular bacteria. The monolayers were washed three times with PBS and 100 μl trypsin-EDTA solution was added to each well. After 5 min, 900 μl of 1% sterile saponin was added and the lysates were plated in triplicates on blood agar and incubated at 37°C for 24 h. The number of CFU was determined at 90 and 210 min post-infection of the cells and expressed as percentage of intracellular bacterial survival after 2 h. Thus, one hundred percent indicates that no difference in intracellular CFU was detected after two hours. The experiments were repeated three times.

### Computational analysis

Prediction of localization and topology of ArcD was performed using the SignalP 4.1 Server and the TMHMM Server v. 2.0 available at: http://www.cbs.dtu.dk.

## Results

### The *s. suis* ADS is transcribed in five transcriptional units

**Figure 1:**
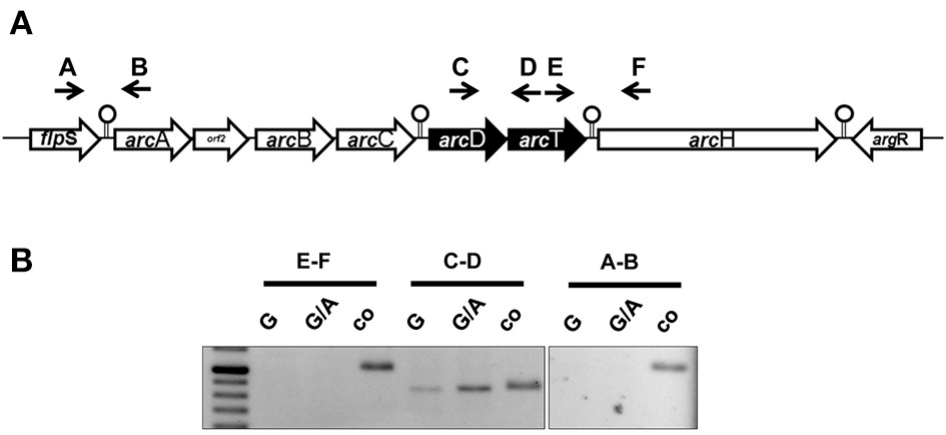
**The *S. suis* ADS is transcribed in five transcriptional units. (A)** Schematic representation of the ADS of *S. suis*. Genes are depicted as arrows pointing toward their transcriptional orientation. Intergenic primers were enumerated with capitals. Stemloop structures separate single transcriptional units. **(B)** Semi-quantitative RT-PCR analysis of *S. suis* WT strain 10 grown in a tryptone-based medium supplemented with 10 mM glucose (G) or 10 mM galactose (G/A) and 50 mM arginine, respectively. Chromosomal DNA was used as positive control. Capitals indicate the intergenic primers depicted in **(A)**.

The ADS is a highly conserved cluster of seven genes encoding the most important arginine-catabolizing pathway in *S. suis* and two flanking genes encoding for transcriptional regulators (Figure 1A, Gruening et al., 2006). The core ADS, facilitating the degradation of arginine to ATP, is composed of three genes: *arc*A, encoding for an arginine deiminase; *arc*B, an ornithine-carbamoyltransferase, and *arc*C, a carbamate kinase. These genes are in close proximity to the putative arginine-ornithine antiporter gene *arc*D and a potential Xaa-His dipeptidase gene *arc*T, as well as *arc*H, a putative endo-β-galactosidase C. Thus, we performed RT-PCR analysis using intergenic primer pairs from RNA of bacteria grown under inducing (TY medium supplemented with 50 mM arginine and 10 mM galactose) and repressive (50 mM arginine and 10 mM glucose) conditions. As depicted in Figure 1B, a positive PCR signal indicating polycistronic transcription was only detected for the *arc*D-*arc*T intergenic region (primer pair C/D) indicating expression of *arc*D and *arc*T from an operon. Interestingly, similar to what is known for the *arc*ABC operon (Gruening et al., 2006), the transcription of *arc*DT was significantly increased when galactose was present as the sole carbon source. In contrast, the regulatory gene *flp*S (intergenic primer pair A/B) located upstream of *arc*A as well as the accessory gene *arc*H (primer pair E/F) were transcribed monocistronically.

### ArcD contributes to growth of *s. suis* and environmental pH homeostasis

**Figure 2:**
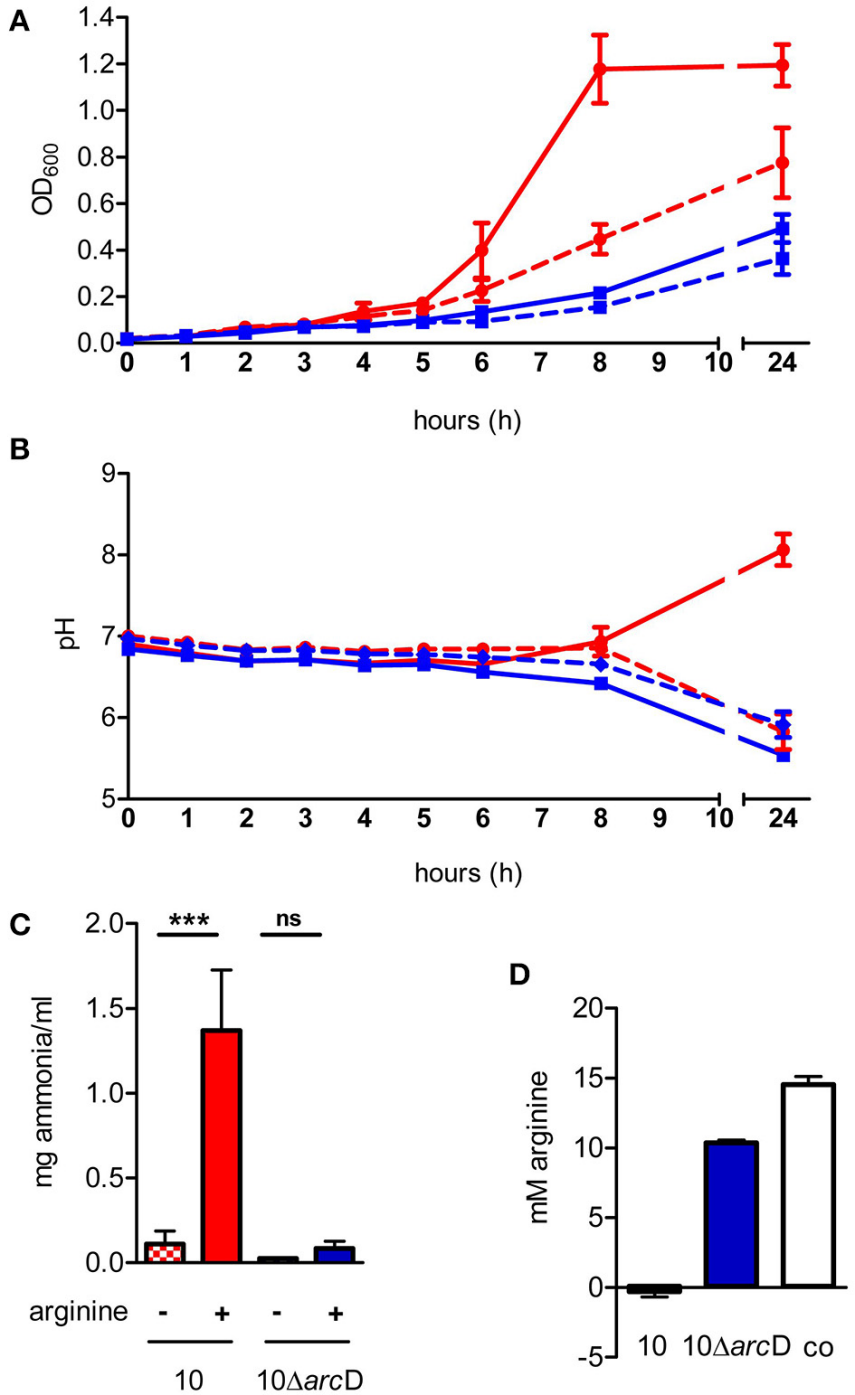
**The *S. suis* ArcD contributes to environmental pH homeostasis and biological fitness in an arginine-dependent manner. (A)** Growth kinetics of *S. suis* WT strain 10 (red) and its isogenic *arc*D-deficient mutant strain 10Δ*arc*D (blue) in a tryptone-based medium containing 10 mM galactose (dotted lines) and 50 mM arginine (solid lines) if indicated. The optical density at 600 nm OD_600_ was measured every hour. Data represent means and standard deviations of a representative experiment performed in triplicates. Experiments were repeated at least two times. **(B)** Bacteria were grown as described in **(A)**, the course of pH values were measured every hour. Data represent means and standard deviations of a representative experiment performed in triplicates. Statistics were performed in a one-tailed *t*-test with *p* < 0.01. **(C)** Ammonia production of the culture supernatant was measured after 24 h of growth. Results are given as mg ammonia per ml of medium (ml^−1^). Statistical significance is indicated for a one-sided *t*-test (^***^p < 0.001; ns, not significant). **(D)** The amount of arginine in the culture supernatant of WT strain 10 (red bar) and 10Δ*arc*D (blue) was detected by a method adapted from Sakaguchi (1925). Results are given in mM arginine. TY medium alone (white bar) served as negative control.

*In silico* analysis of the *S. suis* ArcD revealed significant homologies to transmembranal proteins with arginine-ornithine antiporter function of other streptococci and other arginine-fermenting bacteria (Table 1). However, functional studies on this topic are rare. Therefore, we inactivated *arc*D by insertion mutagenesis and characterized the phenotype of the mutant by growth kinetics. As depicted in Figure 2A, a comparable growth of WT strain 10 and its isogenic mutant strain 10Δ*arc*D was observed in the first hours of growth with a mean OD_600_ ranging between 0.0279 ± 0.008 (WT, with arginine supplementation) and 0.0315 ± 0.0055 (10Δ*arc*D, without arginine supplementation). After 4 h, the growth of WT strain 10 (red lines) was higher as compared to the *arc*D-deficient mutant strain (blue lines). Interestingly, supplementation of arginine (solid lines) did not lead to a higher growth, neither of WT strain 10 nor of the mutant until 5 h. After 6 h, WT strain 10 reached an OD_600_ of 0.2253 ± 0.047 (broken red line) without arginine supplementation, and an OD_600_ of 0.3983 ± 0.12 (solid red line) when arginine had been supplemented. In contrast, significantly lower OD values were detected for the *arc*D-deficient mutant strain, with (0.1343 ± 0.025, solid blue line) and without arginine supplementation (0.093 ± 0.001, broken blue line). Nevertheless, though less prominent the mutant strain 10Δ*arc*D showed an arginine-dependent phenotype similar to the WT strain. Overall, differences in bacterial numbers and arginine availability increased over time between both strains.

**Table 1:** **Comparison of putative arginine-ornithine transporters of different bacterial species**.

**Species**	**Protein name**	**Identities^a^**	**Transmembranal helices^b^**	**Accession number**
*S. suis*	ArcD	–	13	AAY78938
*S. gordonii*	ArcD	71	12	ABV10292
*S. pneumoniae*	Arginine-ornithine antiporter	68	12	ACA36359
*S. uberis*	C4-dicarboxylate anaerobic carrier protein	64	12	CAR42879
*S. pyogenes*	Arginine-ornithine antiporter	64	13	AAT87428
*S. equi* sub. *zooepidemicus*	ArcD	63	13	ACG91640
*Enterococcus faecium*	C4-dicarboxylate anaerobic carrier, arginine transporter	57	12	EEI60191
*Vibrio parahaemolyticus*	Arginine-ornithine antiporter	44	10	EED27648
*Escherichia. coli*	ArcD	44	13	YP_001816563
*Pseudomonas aeruginosa*	ArcD	25	13	AAA25719

a Based on ArcD of S. suis (accession number: AAY78938).

b Prediction, as evaluated by TMHMM.

Next we monitored changes in the pH of the medium during growth of WT strain 10 and its *arc*D-deficient mutant. As depicted in Figure 2B, pH values of the culture medium decreased similarly for strain 10 and 10Δ*arc*D without arginine supplementation. Nevertheless, a slight difference between strain 10 (6.855 ± 0.065) and 10Δ*arc*D (6.655 ± 0.005) was detected at 8 h of growth. This difference was even more prominent when external arginine was supplemented to the growth medium (solid lines). WT strain 10 was able to antagonize growth-dependent acidification of the culture medium resulting in an increase in pH from 6.905 ± 0.059 at the time of inoculation to 8.063 ± 0.2 (solid red line) after 24 h, whereas the pH values detected for the *arc*D-deficient strain (blue lines) dropped similarly to those monitored without arginine supplementation from 6.8425 ± 0.03 (0 h) to 5.54 ± 0.07 (24 h).

Our previous studies showed that ADS-dependent ammonia production as a by-product of arginine catabolism is essentially involved in environmental pH homeostasis (Fulde et al., 2011). To investigate whether similar effects hold true for the ArcD-deficient mutant strain, we determined ammonia production of WT strain 10 and its *arc*D-deficient mutant strain grown under conditions with and without supplementation of arginine (Figure 2C). As expected, supplementation led to a more than 10-fold increase of ammonia production (0.11 ± 0.08 mg ml^−1^ vs. 1.37 ± 0.36 mg ml^−1^) for WT strain 10. Interestingly, although strain 10Δ*arc*D was also able to increase the amount of ammonia in the presence of arginine (0.084 ± 0.04 mg ml^−1^ vs. 0.026 ± 0.001 mg ml^−1^), this effect was comparable to that seen in growth of WT strain without arginine supplementation.

Since ArcD is predicted to be an arginine-ornithine antiporter, we wondered whether a deletion in the respective gene would lead to deficiencies in arginine uptake. For this, we adapted the method described by Sakaguchi (1925) to determine extracellular arginine concentrations. As depicted in Figure 2D, WT strain 10 was able to completely deplete free arginine from the bacterial culture medium (red bar). In contrast, the medium inoculated with strain 10Δ*arc*D (blue bar) still contained significantly higher amounts of arginine (10.371 ± 0.18 mM) at the same OD.

In summary, these results indicate that ArcD is involved in the arginine uptake which is necessary to support the central functions of the ADS. Furthermore, they show that extracellular arginine is important for bacterial growth and a substrate for the arginine deiminase system in *S. suis*.

### *S. suis* is auxotrophic for arginine provided by ArcD

**Figure 3:**
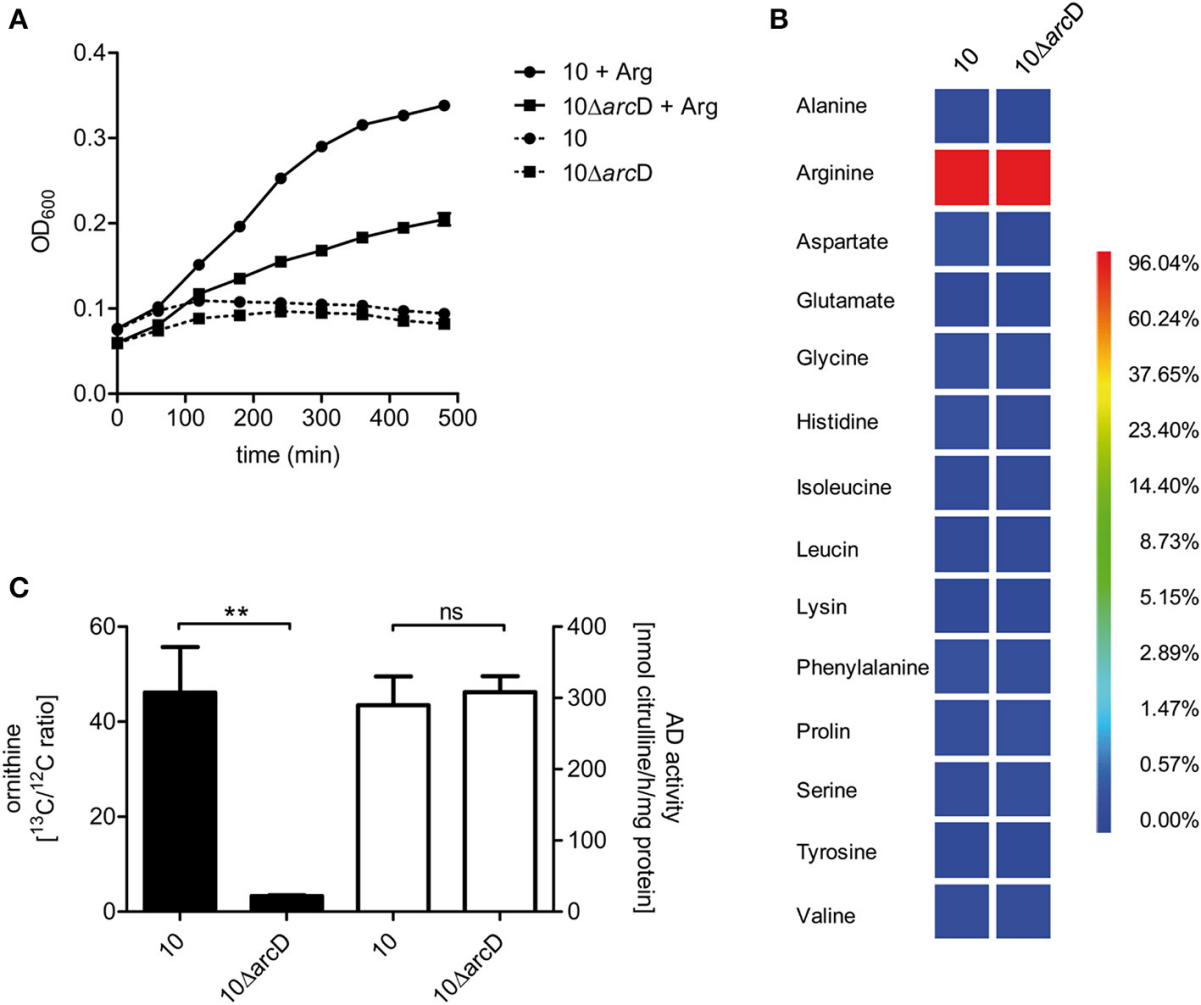
***S. suis* is auxotrophic for arginine and *arc*D contributes to the uptake of extracellular arginine. (A)** Growth of *S. suis* strain 10 and strain 10Δ*arc*D in chemically defined medium (CDM) in which the amino acid arginine was omitted if required. Streptococcal growth was monitored hourly by measuring the optical density at 600 nm (OD_600_). Results are given as mean and standard deviation of one representative experiment performed in triplicates. Assays were repeated at least four times. **(B)** Isotopolog profiles of proteinogenic amino acids after growth of *S. suis* in CDM supplemented with 2.5 mM [U-^13^C_6_]arginine. Multiple ^13^C-labeled isotopologs were determined by GC/MS spectroscopy and the overall ^13^C excess (%) of labeled isotopologs is shown in the color map. Results are shown for one representative experiment. **(C)** Measurement of ^13^C incorporation in ornithine after [U-^13^C_6_]arginine uptake of *S. suis*. After growth in CDM with ^12^C-arginine, *S. suis* strain 10 and strain 10Δ*arc*D were incubated in CDM supplemented with 2.5 mM [U-^13^C_6_]arginine for 30 min. The ^13^C/^12^C ratio of intracellular ornithine was determined as an indirect measure of arginine uptake (black bars, left y-axis). No difference in arginine deiminase (AD) enzymatic activity was observed for both strains under these conditions (white bars, right y-axis). Results are given as mean and standard deviation of three independent experiments. Statistical significance is indicated for a two-sided *t*-test (^**^p < 0.01; ns, not significant).

In order to demonstrate the contribution of ArcD to arginine uptake we performed growth experiments in a chemically defined medium (CDM) containing all amino acids including or excluding arginine. These experiments revealed that *S. suis* strain 10 and strain 10Δ*arc*D were not able to grow in CDM medium containing all amino acids except arginine (Figure 3A). Supplementation of arginine restored growth of both strains, even though the growth of strain 10Δ*arc*D was remarkably diminished when compared to that of the parental strain. These data indicate that *S. suis* strain 10 is auxotrophic for arginine and that ArcD contributes to arginine uptake.

In order to verify that *S. suis* is capable to take up arginine by ArcD and incorporate arginine in newly synthesized proteins, we performed labeling experiments in CDM supplemented with [U-^13^C_6_]arginine that were followed by the detection of the ^13^C-label in protein derived amino acids by GC/MS analysis. As depicted in Figure 3B, [U-^13^C_6_]arginine was taken up and used for protein biosynthesis in both strains. It is important to note that in this experiment the ^13^C excess in arginine is no quantitative value for uptake, because [U−^13^C_6_]arginine is the sole arginine source which is also taken up by the *arc*D-deficient strain (Figure 3A). However, high levels of ^13^C-enrichment were not detected in any other proteinogenic amino acid excluding *de novo* biosynthesis of these amino acids from [U-^13^C_6_]arginine as a precursor. Nevertheless, ^13^C-excess below 1 mol% was found as a ^13^C_1_-labeled isotopolog in aspartate. This indicates that ^13^CO_2_, formed as a by-product of ADS mediated [U-^13^C_6_]arginine catabolization, is used as a precursor in a carboxylation reaction required for aspartate biosynthesis. However, the overall ^13^C excess in arginine did not differ between the WT strain and strain 10Δ*arc*D. Since bacteria were harvested at the same optical density this may be an explanation for that, and these results emphasize that arginine uptake is the growth limiting step for strain 10Δ*arc*D in CDM.

To further elucidate if 10Δ*arc*D has a reduced capacity to take up arginine, we reduced the [U-^13^C_6_]arginine labeling time to 30 min and determined the ^13^C/^12^C ratio of free intracellular ornithine, a product of the arginine deiminase pathway, since the free arginine levels were under the detection limit. The efficiency of [U-^13^C_6_]arginine derived ^13^C incorporation in intracellular [U-^13^C_5_]ornithine was approximately 15-fold higher in the WT strain when compared to strain 10Δ*arc*D (Figure 3C, left panel). The arginine deiminase activity did not differ between both strains under these conditions (Figure 3C, right panel) which excluded a different arginine consumption of the strain. Taken together, these results indicate that ArcD is an arginine transporter.

### ArcD facilitates survival under acidic conditions

**Figure 4:**
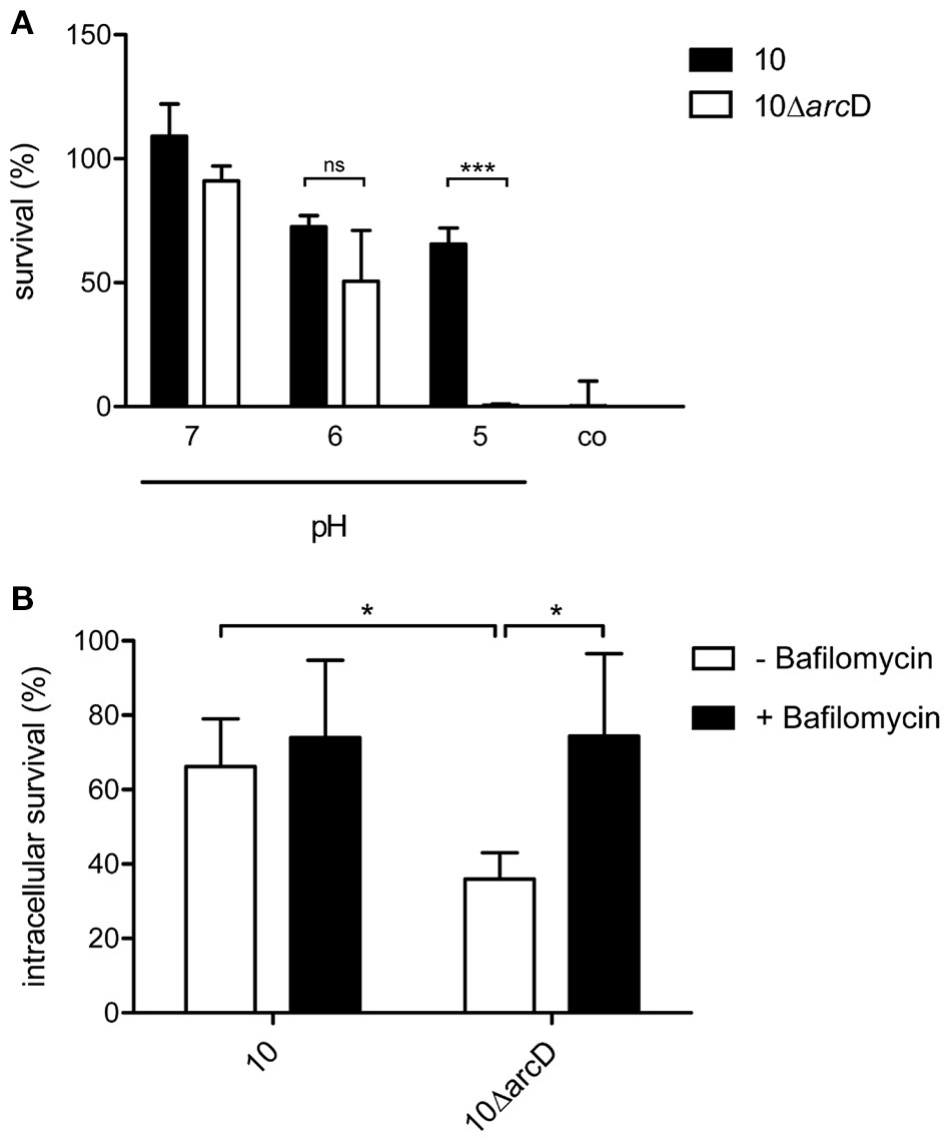
**ArcD facilitates survival under acidic conditions. (A)** *S. suis* strain 10 (black bars) and 10Δ*arc*D (white bars) were incubated for 4 h in a phosphate buffer supplemented with arginine and adjusted to the depicted pH values. Acidic resistance was monitored by replica-plating. Results are given as percentage of the inoculum. Phosphate buffer adjusted to pH 5 without arginine supplementation served as negative control. Data represent means and standard deviations of a representative experiment performed in triplicates. Experiments were repeated at least four times. **(B)** Intracellular survival of the wild-type strain 10 and strain 10Δ*arc*D in HEp-2 cells that were treated with bafilomycin (200 nM) for 1 h before infection (black bars) and in untreated cells (white bars). Results are given as percentage of intracellular bacterial survival after 2 h. Data represent means and standard deviation of three independent experiments. Results were considered statistically significant with *p* < 0.05 in a one-tailed *t*-test, as indicated by asterisks.

The above data indicate a central role of arginine and arginine uptake for the metabolism of *S. suis.* Therefore, we next analyzed the relevance of ArcD for bacterial survival. As shown in Figure 4A survival of WT strain 10 (black bars) and its isogenic, *arcD-deficient* mutant strain 10Δ*arc*D (white bars) differed when incubated in an arginine-containing buffer with pH values adjusted to 5.0, 6.0, and 7.0, respectively. Bacteria were replica-plated after 4 h to monitor survival. No significant differences were observed at pH values of 7.0 and 6.0. However, at pH 5.0, the survival rate of the WT strain was 65.5% ± 6.5, whereas the *arc*D-deficient mutant strain was almost completely killed (0.5% ± 0.5). As a control, bacteria were incubated in buffer adjusted to pH 5.0 without the supplementation of arginine. Under these conditions, strain 10 and 10Δ*arc*D were similarly affected in survival emphasizing the important role of ArcD as an arginine supplier for ADS-mediated resistance under acidic conditions.

In order to elucidate the relevance of ArcD for biological fitness of *S. suis* in a biological model, we performed infection experiments with the epithelial cell line HEp-2. As shown in Figure 4B, the WT strain 10 was able to survive intracellularly at a rate of about 70%, whereas significantly lower survival rates (approximately 35%) were determined for the *arc*D mutant strain. To analyse whether reduced survival correlated with acidification and, thus, the inability of strain 10Δ*arc*D for efficient arginine supply to generate ammonia via the ADS and prevent acidification, HEp-2 cells were treated with bafilomycin to inhibit endosomal acidification before infection. Compared with the infection of untreated cells, the pretreatment of the cells with bafilomycin significantly increased the survival rate of strain 10Δ*arc*D. These data suggest that ArcD substantially contributes to efficient arginine uptake in *S. suis* and, thereby, to its resistance against endosomal acidification in HEp-2 cells.

## Discussion

Streptococci are characterized by a small genome size of approximately 2 Mbp and a homofermentative metabolism with the glycolysis as the primary energy providing pathway (Hoskins et al., 2001; Tettelin et al., 2002; Yamamoto et al., 2005). It is well established that the ADS and the catabolism of arginine is important in metabolism and virulence. The impact of substrate uptake and supply, however, remains mostly elusive. This study focussed on the characterization of ArcD, a putative arginine-ornithine antiporter, located in the gene cluster of *S. suis* ADS. An association of *arc*D to the genes of the ADS is common among different bacterial species, but the genetic organization varies substantially. For example, in *S. suis, arc*D is located downstream of *arc*C and closely associated to the putative Xaa-His-dipeptidase *arc*T (Zuniga et al., 2002; Gruening et al., 2006; Hitzmann et al., 2013). Such an intimate and conserved occurrence of genes often indicates a functional relation of the respective proteins. Indeed, our RT-PCR analysis using intergenic primers revealed that *arc*D and *arc*T are transcribed from a single RNA, the *arc*DT operon, which is separated from *arc*ABC but co-regulated. Yet, the function of *arc*T has not been proven experimentally, though ArcT is a predicted dipeptidase which might provide arginine from oligopeptides.

In order to get more insights into the role of ArcD in *S. suis* metabolism and virulence, we inactivated the respective gene by insertion mutagenesis. Phenotypic characterization was done by growth experiments under ADS inducing conditions. Thus, we used a tryptone-based medium with galactose as the sole carbon source. By this, in contrast to glucose, the ADS is relieved from carbon catabolite repression (CCR). We hypothesized that if ArcD is an antiporter facilitating arginine uptake, phenotypic differences between the WT and the *arc*D negative mutant strain might be more pronounced under ADS inducing conditions, since a substantial contribution of the *arc*ABC operon to the biological fitness of *S. suis* was indicated from our previous studies (Gruening et al., 2006; Fulde et al., 2011). Indeed, strain 10Δ*arc*D was markedly hampered in growth and this effect occurred at very early growth times. Supplementation of the growth medium with arginine led to an increase in growth in both strains but could not compensate the growth defect of the *arc*D mutant. These results suggested a particular role of arginine for growth of *S. suis* and a contribution of ArcD to arginine uptake. Thus, arginine supplied by ArcD seemed to be responsible for the enhanced growth of WT strain 10 under ADS inducing conditions. To further confirm these observations, we repeated the growth experiments in a standardized chemically defined medium to exclude effects mediated by the use of tryptone (Figure 3A). Interestingly, without arginine supplementation, neither WT strain 10 nor the *arc*D deficient mutant strain were able to grow, demonstrating the essentiality of arginine. In turn, supplementation of arginine enabled both the WT strain 10 and its mutant 10Δ*arc*D to grow in CDM, although the growth of the mutant strain was significantly attenuated. This further indicated that ArcD has a considerable impact on the uptake of arginine. Indeed, by using labeled [U-^13^C_6_]arginine, the ^13^C/^12^C ratio of intracellular ornithine was determined to be higher in the WT strain than in its *arc*D deficient derivative. Importantly, this phenotype is not due to a reduced AD activity, as demonstrated in Figure 3C, but to a diminished arginine uptake. This is also shown by an additional independent technique. The comparison of the amount of arginine in the supernatant of bacterial cultures was monitored colorimetrically and revealed significant lower levels in those of the WT strain (Figure 2D).

Arginine auxotrophy was also shown for other streptococci such as *S. canis* and *S. pneumoniae*, respectively (Kloosterman and Kuipers, 2011; Hitzmann et al., 2013). It is well known that streptococci must acquire many nutrients since *de novo* synthesis of metabolic intermediates is restricted due to the small genome size. Thus, closely related *S. pneumoniae* and *S. agalactiae* strains express up to four different arginine uptake systems under starvation conditions (Bryan et al., 2008; Kloosterman and Kuipers, 2011). It is, therefore, conceivable that in *S. suis* alternative arginine providing systems exist which enable the pathogen to multiply even without ArcD. Nevertheless, *arc*DT is closely localized to the *arc*ABC operon and the considerable impact for the growth of *S. suis* substantiates its importance under arginine limited conditions which might be present in different host comportments such as the phagosomal vacuole. We have previously shown that *S. suis* resides in acidified phagolysosome-like compartments after uptake into HEp-2 cells and that ammonia production by ADS-mediated arginine catabolism significantly contributes to intracellular survival of *S. suis* (Benga et al., 2004; Fulde et al., 2011). In agreement, our present data clearly show that a lack in *arc*D leads to a significant reduction in the biological fitness of *S. suis*. The mutant strain was hampered in counteracting environmental acidification and to survive in epithelial cells, which are target host cells during infection.

**Figure 5:**
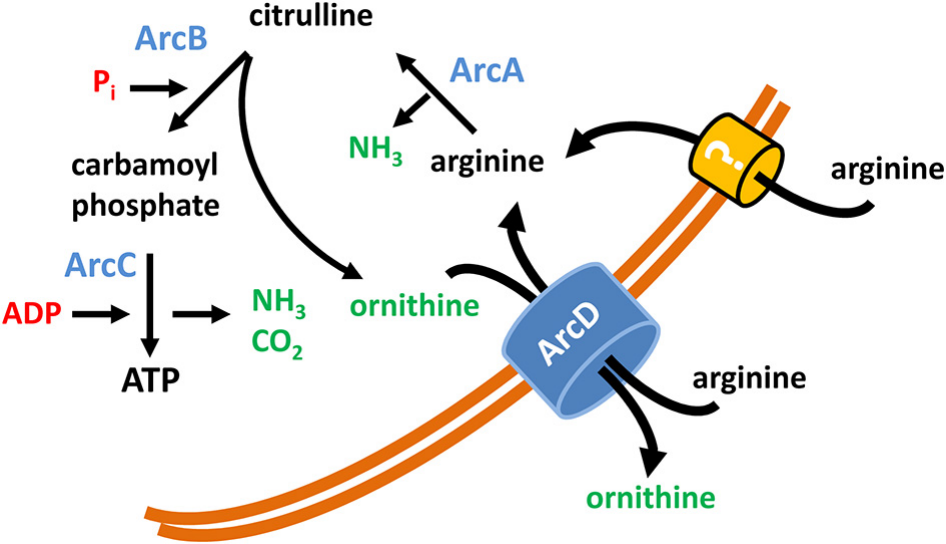
**Model of the function of ArcD as part of the *S. suis* ADS**. The core ADS enzymes (ArcA, ArcB, ArcC) facilitating the conversion from arginine to ornithine are depicted in blue. Metabolic intermediates are indicated in black. The input of energy in terms of phosphate derivatives (P_i_, ADP) are marked in red, non-catabolized and excreted products have a green color. ArcD, located in the bacterial membrane, facilitates an arginine/ornithine antiport. The occurrence of an alternative arginine transporter is illustrated by a question mark.

In conclusion our data clearly denote an important role of arginine and arginine uptake executed by ArcD for the metabolism and survival of *S. suis* (summarized in a model as depicted in Figure 5). Furthermore, they emphasize the outstanding importance of the ADS for biological fitness and pathogenic potential for zoonotic *S. suis*.

## Author contributions

Peter Valentin-Weigand, Marcus Fulde, Joerg Willenborg, and Ralph Goethe designed research; Marcus Fulde, Joerg Willenborg, Daniela Willms, Angela Hitzmann, and Maren Seitz performed the experiments and analyses; Claudia Huber and Wolfgang Eisenreich performed the isotopolog profiling experiments and analyses; and Marcus Fulde, Joerg Willenborg, Ralph Goethe and Peter Valentin-Weigand wrote the paper.

### Conflict of interest statement

The authors declare that the research was conducted in the absence of any commercial or financial relationships that could be construed as a potential conflict of interest.
